# Prohibition—Its Definition, Object, Feasibility and Consequences, with Other Considerations of the Subject, from the Standpoint of a Physician

**Published:** 1904-11

**Authors:** C. H. Wilkinson

**Affiliations:** San Antonio, Texas


For Texas Medical Journal.

### Prohibition—Its Definition, Object, Feasibility and Consequences, With Other Considerations of the Subject, From the Stand-point of a Physician.

BY C. H. WILKINSON, M. D., SAN ANTONIO, TEXAS.

Prohibition, as undertsood in its application to the people of this State, means the banishment of every form of alcohol from Texas, except such varieties as may be permitted to remain in the drug stores for medical purposes only, wines for religious usages, and certain patented preparations occupying an intermediate position between medicine and alcoholic beverages. It means that whisky, brandy, gin, rum, all varieties of wines, cordials, bitters, ale, beer, porter, etc., except those articles above excluded shall be prohibited from sale, and that whoever engages in their traffic or exposes them for sale will be guilty of a felony, and be punishable by fine.

To this end a party has sprung up in Texas and elsewhere, known as the Prohibitionists, and a spirit has developed amongst them which promises to attain a great degree of fervor before the issue presented has been settled.

The subject is a complex one, and the interest of every man, woman, and child in the State is involved in its outcome.

Prohibition is a debatable subject from many standpoints. It has its moral, economical, commercial, and physiological or medical aspect, and naturally draws into its discussion many and varied interests and callings. It is considered a political question, since it has entered the arena and become a bidder for the votes of the people; but, as stated above, so many and so varied are the features entering into its composition, that its discussion is pertinent by nearly every class of our citizenship, and by none with more propriety than by the medical profession.

My remarks, however, will be confined as nearly as possible to the medical features of prohibition, leaving the moral and other aspects of the subject to those better qualified to discuss them.

The fact that prohibition is a political question, however, should not debar the profession of medicine from its discussion, but rather it should act as an incentive thereto, in as much as a great physiological principle is involved in its issue, and one of the great aids to therapeutics is on trial for its existence in the State.

Besides this, every public-spirited citizen, be he doctor, clergyman, merchant, or of any other calling, should feel deep interest in whatever topic concerns the people of his neighborhood, and should ever be willing and ready to lend his voice and his means, if necessary, in promoting their mental, moral, and material welfare.

The objects of prohibition are ostensibly and avowedly to diminish inebriety, and, thereby, crime, pauperism, and unhappiness, and to substitute temperance, virtue, and contentment in the communities in which it prevails.

In this respect the intention of its advocates is most commendable, and surely no reasonable being in the State of Texas exists who does not wish them to succeed. Every adult in this State is unfortunately more or less familiar with the external effects of alcohol upon the human system. There are other effects, however, of this powerful agent for evil which are invisible to the ordinary observer, yet easily recognized by the experienced physician, which are as blighting to the victim of its abuse as his external manifestations are to those he comes in contact with.

Prohibition seeks to counteract the pernicious invisible as well as the visible effects of alcohol upon the human being.

Is prohibition capable of effecting this change in mankind by the banishment of alcohol from a given community, is the question now before us. Is it feasible? Is it practicable? Does it prevent the consumption of strong liquors by those habituated to their use? These are questions which will be left unanswered until the drinking habits of man have been investigated.

As far back as history carries us man has been accustomed to the use of stimulating beverages. How or why he acquired his taste for alcohol is simply a matter of conjecture. Suffice it to say that, for 4000 years or more he has indulged his appetite in this direction, and has continued such indulgence down to the present day.

In the beginning his libations were confined to wine, later on to wine and beer, but with the discovery and development of this country came the establishment of great distilleries, and his taste soon expanded so as to include their products, the strongest form of alcoholics used for drink.

It is worthy of note, however, that all through ancient and mediæval times the beverages of those periods were looked upon with favor, and even deities were dedicated to their use. Neither crime nor pauperism were attributed to their abuse, and mankind was happy and healthy in their free and unrestricted enjoyment.

It was not until the distiller began his work that gross inebriety, vice and pauperism, as a consequence of alcohol, began to appear.

Thus it would seem that man's habits of drink were the evolution of centuries, and that they are, consequently, fixed, and irremovable by any sudden change that might be suggested by his fellow-beings. To him alcohol in some shape or other is a prop and a necessity, without which his energy would lapse and his vital forces flag. The habitue, given his choice of stimulants, naturally chooses the stronger, for the simple reasons that they are much quicker in bringing about their exhilarating effects, and produce a higher degree of stimulation when taken into the system. In the absence of his stronger drinks, however, he will reluctantly, though speedily, turn to milder and less harmful ones, and in the course of time will substitute the latter for the first.

Deprived of both, however, the habitue of alcohol immediately casts about to procure the strongest class of drinks, regardless of law, the purity of his liquor, or of other consequences, thereby defeating the primary object of prohibition.

These statements are facts which can be abundantly verified by the testimony of many good physicians residing in local option precincts, and tend to answer, in the *negative,* all the interrogatories asked above concerning the feasibility of prohibition.

### THE NECESSITY FOR ALCOHOL.

The necessity for stimulants of some kind, by mankind, has been recognized from time immemorial by the medical profession.

Until the rectifying of spirits came into vogue, wine was used freely, not only as a true medical agent, but to the aged, the feeble, and the weary it has always been advocated without stint, both to the enjoyment and benefit of the user.

The Bible itself, the highest exponent of morality and temperance known to man, abounds in passages extolling the virtues of and the necessities for wine. It is a pertinent question, indeed, to inquire why alcohol was given to man, as the human race is the only one on earth adopting it for any purpose whatever. In the great order of creation it stands to reason that it was created and ordained for his exclusive enjoyment and use. Nothing created is aimless and superfluous.

To assume that the enormous products of the vineyards and the fields were given to the world for purely medicinal purposes is an unreasonable supposition. It is far more likely that all forms of alcohol were given to man for his refreshment, and that he is held accountable by the Giver for their moderate and proper use. The mischief lies not so much in the agent as in the actor. Alcohol, in its strongest form, is unquestionably the cause of much unhappiness and crime. So are other deadly agents, including gunpowder.

Man is a complex creature with a body and a mind. He is subject to fatigue, worry and mental taxation. There are times in his existence when a mild stimulant is as essential for him as the food he eats.

To the aged, the weary and the careworn, there is no question of the necessity for alcohol, whether such be invalids or not. Not to excess, nor in adulterated qualities, but in moderation at all times, and in the purest form it can be taken.

To the invalid it is invaluable, and for convalescence it is almost indispensable.

Carpenter, the great English author, whose work on physiology is now a standard text-book in colleges and universities all over the world, says this of alcohol: "It is certain that alcohol is not without its value under various corporeal conditions, and the views expressed by Dr. Hammond that its effects, injurious or salutary, are in a great measure dependent upon the quantity of food consumed with it, are probably true.

When the food is ample in quantity and varied in quality, and the digestion good, alcohol is unnecessary, but when the diet is insufficient, or the digestion feeble, the effects of alcohol, when taken in moderate quantities, seem decidedly beneficial, the body not only ceasing to lose but actually gaining in weight, the alcohol either taking the place of the food or retarding the metamorphosis of the tissues. In conditions of exhaustion it often proves of the greatest utility, and there is reason for believing that it can exert an antiseptic power in certain states of blood poisoning, whilst it exerts a powerful influence over the formation of pus in the inflamed parts." Page 118.

That the habit of strong drink is conducive to ill-health and other evils is true; that some restrictions to its unlimited use is necessary, I believe; but that the banishment of every variety of it from any community will effectually cure intemperance, I doubt; and that the moderate use of light wines and pure beer, by the public, is detrimental to their health, I emphatically deny. Indeed, as far as temperance is concerned, could we return to mediæval ages, where wine and song were almost synonymous, when gross intoxication was never known, and crime as a consequence of alcohol was almost never heard of, we would be better off and happier today.

If sobriety must be acquired by law, then let us adopt it through some rational, lasting and satisfactory manner. The writer has spent hours amongst our German citizens, on the occasion of their festivals. He has seen thousands of them congregated where their national beverage flowed like water. Yet, not a single case of intoxication was noticed. The occurrence struck him as remarkable and un-American, but upon investigation, when the nature of the drink was inquired into, and only 5 per cent of alcohol found in the beer they were drinking, then his wonderment was changed into admiration for a beverage that could afford so much refreshment and exhilaration to the masses, without producing some evidences of intoxication. If one wishes to see communities practically free from inebriety, and specimens of physical manhood that can not be surpassed anywhere in this country, he should go into the German villages and towns of Texas and note the character of those communities in these respects. Every one drinks beer who drinks stimulants at all, yet inebriety is rarely ever seen.

Only recently it was announced through the public press that the Governor of Sonora, Mexico, had directed that beer be used to supplant the ordinary stimulating beverages used by the natives of his State, having discovered that for general drinking purposes it was preferable for the masses, as inducing less intoxication than the usual native drinks.

This is a remarkable indorsement for beer, as well as a remarkable remedy for inebriety, yet it tends to confirm an opinion long entertained by the writer, that if you induce the employment of a mild grade of stimulants, by the masses—putting the stronger ones aside—their taste will eventually adopt the former to the total exclusion of the last.

The practice of substituting a harmless for a harmful stimulant would answer all the reasonable demands of the human system, and might with propriety be tried in Texas.

Only a few weeks since, at a convention of the army and navy surgeons of the United States, it was agreed amongst them to petition the department to reinstate the army canteen, and allow the troops the privilege of supplying themselves with beer, the innovation having proved satisfactory where tried in other places, and to the improvement of the men.

When the Governor of a Mexican State, and the surgeons of the

American army ask for beer as a beverage for those under their jurisdiction, it would seem a waste of time on our part to attempt its banishment from Texas, on account of any fancied injurious influence it might exert over the people.

The writer does not believe that the habit of drinking beer and light wines, in moderation, induces a desire for stronger drinks, amongst those individuals who habitually use them, but on the contrary, like the Governor of Sonora, he believes that a substitution of taste from the stronger for the milder form of alcohol would easily be brought about, especially if the former were placed where it was not convenient to obtain them, and in this substitution of one form for the other, the milder for the stronger, he considers the solution of the temperance question in Texas can be found.

### THE CONSEQUENCES OF PROHIBITION.

Should the prohibition movement prevail in Texas, the following results might reasonably be expected. These conclusions are based upon good reasons, and are confirmed by the testimony of many physicians of experience in the State, who have resided in Prohibition districts, and whose testimony can be relied on.

First. The movement, if successful, would have the effect of depriving the masses, and particularly the poorer people of a much-needed, mild, harmless and at times a necessary tonic, by placing beer and light wines beyond their reach.

Second. To stimulate, amongst certain classes, clandestine methods for procuring whisky from outside sources, the evasion of the laws of the State, and the conversion of private residences into private bar rooms.

Third. To increase the consumption of patent medicines containing the cheaper and more damaging forms of alcohol, as well as that of outside liquors compounded of vile adulterants, and far more dangerous to the health of the people than the brands usually sold to them in the open saloon.

Fourth. The increase of intemperance in the private circle and the club, by introducing into both of them whisky by the gallon, instead of by the drink.

Fifth. To overload and disgust the average medical practitioner with importunities and threats for liquor prescriptions, by the bibulous friend, the unscrupulous toper, and the pleading and suffering victim of its abuse.

Sixth. The embarrassment of the home breweries, if not their actual suppression in the State, by forbidding the sale of their products, and by encouraging their foreign business rivals—the distilleries.

Seventh. By effecting the supremacy of the outside distillers —the surmised power behind the throne of Prohibition, through the destruction of their business rivals, the brewers.

